# Immunomorphological Analysis of the CD40–CD154 Interaction in T Follicular Helper Cell Lymphoma Emphasizes the Significance of the CD40–CD154 Axis in the Disease

**DOI:** 10.3390/cells15090785

**Published:** 2026-04-26

**Authors:** Dóra Mária Janotka, Zita Borbényi, Klára Piukovics, Mátyás Bukva, Annamária Bakos, Enikő Bagdi, László Krenács

**Affiliations:** 1 Laboratory of Tumor Pathology and Molecular Diagnostics, 6726 Szeged, Hungary; 2 Pathology Department, Semmelweis Hospital, 6400 Kiskunhalas, Hungary; 3 Department of Hematology, Internal Medicine Clinic South Division, Albert Szent-Györgyi Clinical Center, University of Szeged, 6725 Szeged, Hungary; 4 Department of Immunology, Albert Szent-Györgyi Medical School, University of Szeged, 6725 Szeged, Hungary; 5 Department of Nuclear Medicine, Albert Szent-Györgyi Clinical Center, University of Szeged, 6725 Szeged, Hungary

**Keywords:** nodal peripheral T-cell lymphoma, T-follicular helper cell lymphoma, angioimmunoblastic type, follicular type, NOS type, CD40 and CD154 expression, CD40–CD154 interaction in T-cell lymphoma

## Abstract

**Highlights:**

**What are the main findings?**

- Neoplastic cells in T follicular helper cell lymphomas consistently express CD154.
- The CD154 expression increased in histologically advanced cases and correlated with the extent of CD40-positive cell mass.

**What are the implications of the main findings?**

- CD40–CD154 interaction influences the lymphoma microenvironment.
- CD40–CD154 crosstalk plays a role in lymphoma-associated immune dysregulation.

**Abstract:**

Peripheral T-cell lymphomas (PTCLs) are malignancies of mature T cells with a poor prognosis. Most PTCL cases express follicular T-helper (T_FH_) cell antigens and are classified as T_FH_ cell lymphoma (TFHL). Contact-dependent signaling between CD40 and its ligand, CD154, is essential for immune functions. CD154 is expressed by activated T cells, while CD40 is found on B cells, follicular and other dendritic cells, macrophages, and stromal cells. Although the CD40–CD154 crosstalk is a key costimulatory pathway in immune responses, data on its role in PTCLs are limited. To explore the role of the CD40–CD154 axis in TFHLs, we conducted an in-depth immunomorphological study of 111 PTCL cases, including 93 TFHL cases. We found that neoplastic T cells in TFHL are consistently CD154-positive. The CD154 expression increased in histologically advanced cases and correlated with the extent of CD40 positivity. We showed that CD154-positive neoplastic T cells recapitulate the intranodal migration of normal TFH cells, disrupting and remodeling each functional compartment, thereby explaining the disease-related immune dysfunction. Our findings indicate that pathological CD40–CD154 interaction is a potential driver mechanism in TFHL and offers a promising target for future therapies.

## 1. Introduction

The group of nodal T-follicular helper cell lymphomas (TFHL), which includes cases previously called angioimmunoblastic T-cell lymphoma (AITL) and other peripheral T-cell lymphomas with a T follicular helper cell (TFH) phenotype, comprises clinically aggressive, morphologically diverse systemic mature T-cell malignancies [1,2,3,4,5,6,7]. These primarily affect elderly patients and are characterized by advanced-stage disease, generalized lymphadenopathy, hepatosplenomegaly, and complications resulting from impaired immune function [2,8,9]. Involved lymph nodes display a polymorphic infiltrate containing abnormal T cells, often with pale cytoplasm, that express multiple TFH-related antigens, alongside various B cells, proliferating high endothelial venules (HEVs), and an abnormal follicular dendritic cell (FDC) network [1,6,7,10,11]. Recent lymphoma classification schemes group these lymphomas as a single entity with three subtypes based on their shared phenotypic, clinical, and molecular characteristics: angioimmunoblastic type (TFHL-AI), follicular type (TFHL-F), and not otherwise specified type (TFHL-NOS) [6,7]. TFHL-AI can be further divided into three histological patterns based on the presence of hyperplastic B-cell follicles (TFHL-AI-1), depleted B-cell follicles (TFHL-AI-2), or complete effacement with prominent follicular dendritic cell (FDC) proliferation (TFHL-AI-3) [12].

CD40 is a type I transmembrane receptor in the tumor necrosis factor superfamily, expressed in various cell types, including B cells, dendritic cells, macrophages, fibroblasts, and endothelial cells [13,14]. CD154, the ligand for CD40 [15,16] exists in two functional forms: as a type II homotrimeric transmembrane protein on the surface of activated CD4+ T cells, especially TFH cells [17,18], and in platelets [19], and as an extracellular soluble form that can act as a cytokine [16,20,21]. The interaction between CD40 and CD154, mediated by contact-dependent bidirectional signaling, is essential for the development of cell-dependent humoral immune responses, organ-specific autoimmunity, and protection against infections [13,14,22,23]. Engagement of CD40 by CD154 induces trimeric clustering of CD40 and recruits TNF receptor-associated factors (TRAFs), thereby activating the MAPK and NF-κB pathways [14,24]. Cross-linking of CD40 on B cells triggers B cell clonal expansion, germinal center formation, isotype switching, affinity maturation, the development of long-lived plasma cells, and the generation of memory B cells [14,24]. In dendritic cells involved in T-cell priming, CD40 signaling induces upregulation of MHC class II and the production of inflammatory mediators, thereby prolonging presentation of MHC/antigen complexes [25,26]. Abnormal humoral responses are believed to play a role in autoimmune diseases, and anti-CD154 treatment may block disease development in mouse models [13].

CD40 plays a crucial role in B-cell activation and survival [13,24]; its expression has been observed in nearly all B-cell lymphomas and Hodgkin lymphoma [27,28]. The CD40–CD154 interaction is a key mechanism of T-cell function, and CD154 is highly expressed at the RNA level [29,30,31]; therefore, it has been proposed as a prime factor in the pathogenesis of TFHL [32].

In a few cases, we previously reported that CD154 is expressed on neoplastic T cells in angioimmunoblastic T-cell lymphoma (now TFHL-AI) [33]. Since then, however, limited data have been available on the in vivo expression of CD154 and CD40 proteins in human TFHL [28,34,35]. Inspired by this, we performed an in-depth immunomorphological analysis of a large cohort of cases to simultaneously investigate the presence and distribution of CD154-positive neoplastic T cells and CD40-positive immune cells, with the aim of better understanding the complex microenvironment of TFHLs and, in turn, the disease’s immunobiology.

## 2. Materials and Methods

### 2.1. Patients and Tissue Samples

Archived tissue samples from 111 PTCL cases diagnosed between September 2001 and December 2025 were obtained from the Laboratory of Tumor Pathology and Molecular Diagnostics in Szeged. This cohort comprised 93 TFHL patients (43 females, 50 males) and 18 PTCL NOS patients (10 females, 8 males) without a TFH cell phenotype (non-TFH PTCL NOS). The median age of TFHL patients was 67 years (range 18–90 years), and 95% were at an advanced stage (III-IV). The median age of non-TFH PTCL NOS patients was 54 years (range 25–82 years), and 67% were at an advanced stage (III-IV). The demographic and pathological data for the analyzed cases are summarized in Supplementary Table S1.

Each tissue sample was fixed in 10% (*v*/*v*) neutral buffered formalin and then routinely embedded in paraffin. The collected cases were diagnosed based on clinical information, histomorphology, and immunophenotypic features. During pathological evaluation, we assessed morphological abnormalities, including disruption of lymph node architecture, pathological transformation of the paracortical tissue, characteristic proliferation of post-capillary venules, the appearance and proliferation of atypical T cells expressing T-cell antigens, distortion or depletion of the B-cell compartment, and pathological networks and abnormal expansion of follicular dendritic cells. A case was considered tumor cell-rich if neoplastic T cells were present at high density in any part of the affected lymph node. To support the diagnosis, when sufficient-quality DNA could be extracted from paraffin-embedded tissue, molecular studies were performed to determine T-cell and B-cell clonality and to detect the RHOA G17V point mutation. LK and EB reviewed and reclassified each case based on TFH cell phenotyping and according to current classification schemes [6,7]. Inclusion criteria were the availability of representative paraffin blocks or adequate unstained slides for additional immunohistochemical analysis. Exclusion criteria were inadequate tissue quantity or poor tissue quality, or a doubtful diagnosis during revision.

This series included 93 TFHL cases (6 TFHL-AI-1, 32 TFHL-AI-2, 26 TFHL-AI-3, 6 TFHL-F, and 23 TFHL-NOS), along with 18 PTCL NOS cases without a TFH cell phenotype. Based on the predominant distribution of neoplastic cells, the TFHL-AI-2 cases were further categorized into subtype A (TFHL-AI-2A, mainly intra- and perifollicular) and subtype B (TFHL-AI-2B, mainly extrafollicular) [36]. Considering cytomorphology and architectural changes, the TFHL-NOS cases were divided into subtype A (TFHL-NOS-A, monomorphic without follicles), subtype B (TFHL-NOS-B, polymorphic infiltration with uninvolved atrophic follicles), and subtype C (TFHL-NOS-C, classic polymorphic infiltrate without follicles) [36].

### 2.2. Immunohistochemistry and Evaluation

Immunohistochemical reactions were performed on whole tissue or tissue microarray sections. Briefly, paraffin sections were routinely deparaffinized and heat-treated in an electronic pressure cooker with the appropriate antigen-retrieval buffer. After protein blocking (RE7102, Leica Biosystems/Novocastra Laboratories, Newcastle upon Tyne, UK), the sections were incubated with primary antibodies at room temperature for 60 min. Detection was carried out using the Novolink polymer kit (Leica/Novocastra). For primary goat or rat antibodies, rabbit anti-goat (Agilent Technologies/DAKO Santa Clara, CA, USA) or rabbit anti-rat linker antibodies were used. Each staining was performed on an 8-channel TECAN Freedom Evo 150 liquid-handling platform (Männedorf, Switzerland). All primary antibodies used in the study are listed in Supplementary Table S2.

The T_FH_ cell phenotype of a PTCL case was determined if at least 4 of the 7 applied TFH cell markers (BCL6, CD10, CD134, CXCL13, CXCR5, ICOS, and PD1) were positive in the abnormal T cells, in addition to CD4 [36].

To increase sensitivity and specificity, two anti-human CD40 and two anti-human CD154 antibodies were tested on whole tissue sections from 5 hyperplastic lymph nodes and 3 hyperplastic palatine tonsil samples.

A mouse monoclonal anti-CD40 antibody (Bio-techne/Novus Biologicals, Centennial, CO, USA, NBP2-34488, clone CL1673) and a rabbit polyclonal anti-CD40 antibody (ThermoFisher/Invitrogen Carlsbad, CA, USA, PA-32325) were tested and found to produce identical staining patterns; therefore, they were used interchangeably in the study.

CD154 was detected using two antibodies: a mouse monoclonal antibody (Proiteintech, Rosemont, IL, USA, 66502, clone 1E6D10, immunogen protein sequence: His47-Leu261) and a goat polyclonal antibody (Bio-techne/R&D Systems, Minneapolis, MN, USA, AF617, immunogen protein sequence: Glu108-Leu261). To confirm the specificity of the anti-CD154 antibodies, a blocking experiment was performed by preincubating the goat polyclonal anti-CD154 (0.3 μg/mL) and the mouse monoclonal anti-CD154 (1 μg/mL) primary antibodies with human recombinant sCD154 protein (ThermoFisher/PeproTech, Cranbury, NJ, USA, 310-02, protein sequence: Glu108-Leu261) at concentrations of 3 μg/mL and 5 μg/mL, respectively, before conducting the standard immunohistochemical procedure.

A T-cell lymphoma case was considered CD154-positive if the abnormal T cells and at least 10% of cells in the affected lymph node showed specific immunostaining with at least one of the two CD154 antibodies. A case was considered CD154-negative if the abnormal T cells were negative for both CD154 antibodies. When CD154 positivity could not be confirmed by single immunostaining, multiplex immunofluorescence staining was performed to assess co-expression of CD154 with TFH markers on the abnormal T cells.

### 2.3. Multiple Immunofluorescence Staining

Simultaneous multiple immunofluorescence staining was performed on representative cases of each TFHL type. Case selection was based on tissue quality and strong immunoreactivity for each antigen to be stained.

After appropriate pretreatment, slides were incubated with a cocktail of two or three primary antibodies from goat, rabbit, mouse, or rat. This step was followed by a single incubation with a secondary antibody cocktail consisting of highly cross-absorbed donkey anti-mouse, anti-rabbit, and anti-goat antibodies labeled with Alexa Fluor Plus 647, Alexa Fluor Plus 488, and Alexa Fluor Plus 555, respectively (all from ThermoFisher/Invitrogen). Slides were mounted with ProLong Glass Antifade with NucBlue Stain (ThermoFisher/Invitrogen).

### 2.4. EBER In Situ Hybridization

Epstein-Barr virus (EBV) was detected by EBER in situ hybridization using a blend of custom-made FITC-labeled oligonucleotide probes for EBER1 (5′FAM-TCACCACCCGGGACTTGTACCCGGGACGGG) and EBER2 (5′FAM-TCCTCCCCCGGGACTTGACCTCGGGTCGG). A mouse monoclonal anti-FITC antibody (Thermofisher/Invitrogen, clone 1F8-1E4) was used to detect the hybridized probes.

### 2.5. Statistical Analysis

CD154 expression was assessed using a semiquantitative score. Base values were 1, 2, and 3, representing weak, moderate, and strong staining intensity, respectively. An additional 0.5 or 1 point was added for medium or large cell morphology, respectively, and a tumor cell-rich pattern received an additional 1 point. Thus, the CD154 positivity score ranged from 1 (i.e., small cells with weak positivity) to 5 (i.e., strongly positive large cells with a tumor cell-rich pattern). CD40 expression was estimated as the percentage of CD40-positive area within the affected lymph node tissue.

Because both variables were non-normally distributed and CD154 was measured on an ordinal scale, nonparametric methods were used throughout the analysis.

Marker distributions were summarized by subtype using sample size, median, interquartile range, mean, and standard deviation.

Differences in CD40-positive area and CD154 score across lymphoma subtypes were assessed using the Kruskal-Wallis test. Post hoc pairwise comparisons were performed using Dunn’s test. To assess whether CD40 and CD154 varied in parallel across subtypes, subtype-level medians were compared using Spearman’s rank correlation. Associations between binary CD154 positivity and binary clinicopathologic variables were analyzed using 2 × 2 contingency tables and Fisher’s exact test. Odds ratios with 95% confidence intervals were also calculated.

For multivariable analysis, logistic regression was performed with CD154 positivity as the dependent variable and CD4 positivity and TFHL status as predictors. Due to sparse data and near-complete separation, a Firth penalized logistic regression model was also applied and used for interpretation.

All tests were two-sided, and *p*-values below 0.05 were considered statistically significant. Statistical analyses and figure generation were performed in R (https://www.r-project.org/, accessed on 17 February 2026).

## 3. Results

### 3.1. Assessment of the Specificity of Anti-CD154 Antibodies

**Figure 1:**
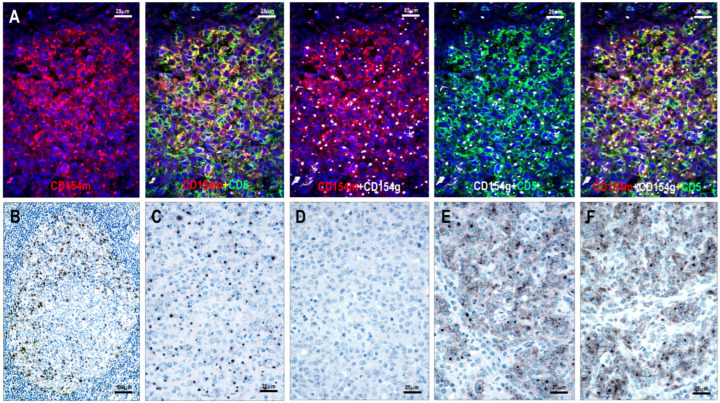
Testing the specificity of anti-CD154 antibodies. (**A**) Colocalization of immunostaining with the mouse monoclonal antibody (CD154m) and the goat polyclonal antibody (CD154g) in CD5-positive T cells (multiplex immunofluorescence labeling, ×400). (**B**) Distribution of CD154-positive TFH cells within a hyperplastic secondary follicle. Positive cells are primarily located in the light zone of the germinal center (magnification ×100). (**C**) Neoplastic T cells in a TFHL-AI-2B case displaying a typical dot-like pattern with the goat polyclonal anti-CD154 antibody (magnification ×400), and (**D**) no immunostaining after blocking the primary antibody with recombinant sCD154 protein (magnification ×400). (**E**) Mouse monoclonal anti-CD154 antibody showing cytoplasmic dot-like and incomplete membrane staining, and (**F**) retained immunoreactivity after blocking with recombinant sCD154 protein (magnification ×400).

Using immunofluorescent multiplex labeling, both CD154 antibodies stained the same T cells (Figure 1). Normal TFH cells generally showed relatively weak cytoplasmic staining, whereas neoplastic TFH cells exhibited cytoplasmic staining with a dot-like condensation in the Golgi region (Figure 1). Additionally, the mouse monoclonal antibody 1E6D10 showed some incomplete membrane staining (Figure 1). Inconsistent weak-to-moderate reactivity was observed in high endothelial venules (HEVs), follicular dendritic cells (FDCs), interdigitating dendritic cells (IDCs), and plasma cells.

The blocking experiment using human recombinant sCD154 protein showed no immunostaining with the polyclonal goat anti-CD154 antibody, whereas the mouse monoclonal antibody retained immunoreactivity (Figure 1). This experiment demonstrated that the polyclonal goat anti-CD154 antibody binds to the extracellular domain sequence Glu108-Leu261, consistent with the soluble protein, whereas the epitope of the mouse monoclonal antibody 1E6D10 lies within the His47-Lys107 sequence.

### 3.2. CD154 and CD40 Staining Pattern in Non-Neoplastic Lymphoid Tissues

In hyperplastic lymphoid tissue samples, CD154 was expressed in intrafollicular TFH cells (Figure 1) and in scattered paracortical T cells. CD40 was strongly expressed in B-cell follicles and IDCs, whereas it was weakly expressed in fibroblastic reticular cells (FRCs). Plasma cells showed no CD40 staining.

### 3.3. CD154 and CD40 Expression in T-Cell Lymphoma Tissues

**Figure 2:**
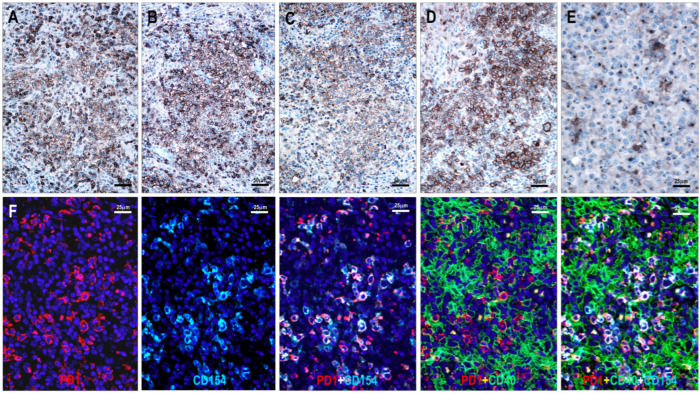
Distribution, immunophenotype, and CD154 expression of neoplastic T cells in a tumor cell-rich TFHL-AI-2A case (**A**–**E**). Atypical medium-sized lymphoid cells show (**A**) CD5, (**B**) CD4, (**C**) PD1, (**D**) ICOS, and (**E**) CD154 positivity; these correspond to neoplastic TFH cells ((**A**–**D**) magnifications ×200; (**E**) magnification ×400). (**F**) PD1-positive neoplastic T cells in a TFHL-NOS-C case co-express CD154 and are intermixed with CD40-positive cells (multiple immunofluorescence labeling, DAPI nuclear staining, ×400 magnification).

**Figure 3:**
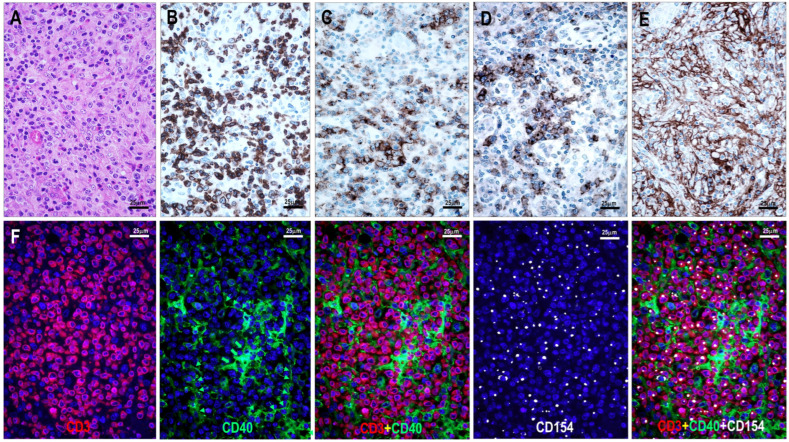
Immunophenotyping of neoplastic T cells with immunoperoxidase in a TFHL-AI-2B (**A**–**E**) and with multiple immunofluorescent staining in a tumor cell-rich TFHL-AI-3 case (**F**). (**A**) Hematoxylin and eosin staining shows the characteristic polymorphic infiltration with medium-sized atypical cells with pale cytoplasm (magnification ×400). The abnormal cells are positive for (**B**) CD3, (**C**) PD1, and (**D**) CD154. (**E**) CD40 shows diffuse positivity, with intermingled CD40-negative, medium-sized neoplastic T cells ((**A**–**E**), magnification ×400). (**F**) A tumor cell-rich area of the same case shows neoplastic T cells co-localizing with CD3 and CD154, in close contact with CD40-positive IDCs and FDCs (multiple immunofluorescent labeling, DAPI nuclear staining, magnification ×400).

**Figure 4:**
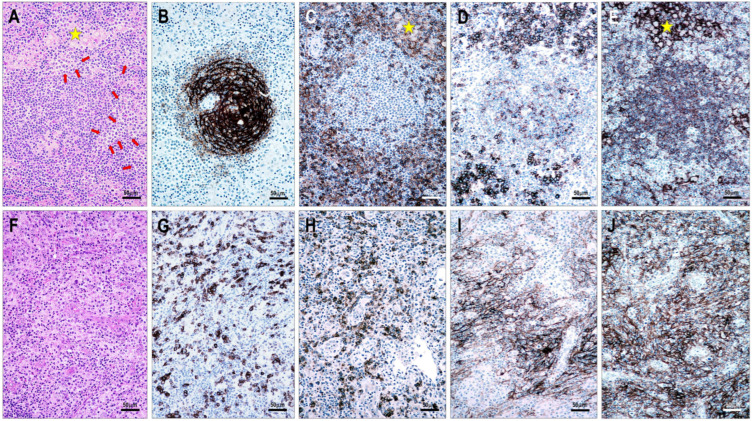
(**A**–**E**) CD40–CD154 status in a case of TFHL-AI-2B and (**F**–**J**) a case of TFHL-AI-3. (**A**) Atypical infiltrate showing groups of medium-sized pale cells (red arrows) and clusters of epithelioid cells (yellow star) (hematoxylin-eosin stain, 200× magnification). (**B**) A CD21-positive dense, concentric FDC network surrounded by (**C**) CD4-positive tumor cells and epithelioid cell cluster (yellow star), and (**D**) CD154-positive tumor cell sheets. (**E**) Residual small B cells in a primary follicle-like aggregate, along with perifollicular epithelioid cells (yellow star) and dendritic cells, are CD40-positive, whereas the neoplastic T-cell sheets are negative ((**A**–**E**), 200× magnification). (**F**) Polymorphic infiltrate in TFHL-AI-3 contains atypical cells with pale cytoplasm surrounding HEVs (hematoxylin-eosin stain, ×200 magnification). (**G**) Atypical cells are CD3-positive, and (**H**) co-express CD154 ((**G**,**E**) magnification ×200). (**I**) Characteristic CD21-positive pathological FDC meshwork and (**J**) diffuse CD40 positivity, extending beyond the CD21-positive FDCs ((**F**–**J**), ×200).

**Figure 5:**
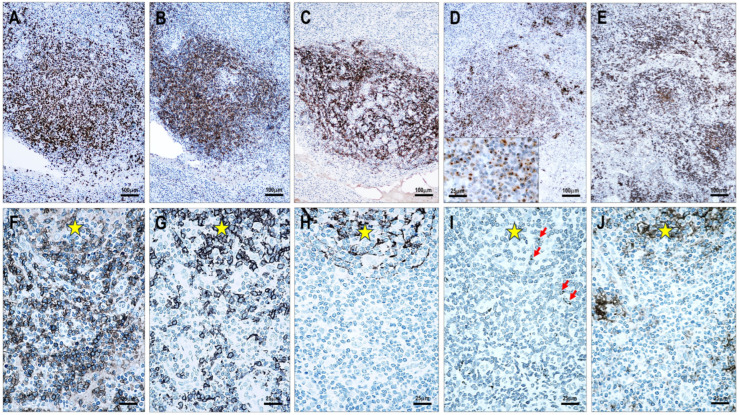
(**A**–**E**) Immunomorphology of TFHL-F. The pathological follicle is filled with (**A**) CD3-positive and (**B**) PD1-positive T cells and shows (**C**) an abnormal CD21-positive network. The abnormal intrafollicular T-cell population is (**D**) CD154-positive. (**E**) CD40 positivity is distributed within and around the follicle ((**A**–**E**) magnifications ×100; (**D**) inset ×1000). (**F**–**J**) Immunomorphology of a non-TFH PTCL case. An atypical T-cell infiltrate surrounding an atrophic follicle (yellow star) shows CD3 positivity (**F**). (**G**) CD20-positive B cells are present within the atrophic follicle (yellow star) and are scattered in the extrafollicular area. (**H**) The follicle (yellow star) shows an atrophic CD21-positive FDC network. (**I**) A few residual small cells are CD154-positive (red arrows). (**J**) A few cells are CD40-positive, mainly associated with the atrophic follicle (yellow star). ((**F**–**J**) magnifications ×400).

Among the 111 lymphoma cases examined, 94 (85%) showed CD154 positivity in neoplastic T cells. All TFHL cases (93/93, 100%) were CD154-positive, including 6/6 (100%) TFHL-AI-1, 32/32 (100%) TFHL-AI-2, 26/26 (100%) TFHL-AI-3, 6/6 (100%) TFHL-F, and 23/23 (100%) TFHL-NOS cases. In contrast, only 1 of 18 (6%) PTCL NOS cases with a non-TFH phenotype showed CD154 positivity (Table 1, Figure 2, Figure 3, Figure 4 and Figure 5).

CD154 positivity colocalized with other TFH cell markers on neoplastic T cells (Figure 2 and Figure 3). In the follicular compartment, the positive cells were located within the abnormal FDC meshwork, adjacent to residual germinal center B cells, and intermingled with mantle zone B cells. In the paracortical compartment, CD154-positive T cells were found in close proximity, often surrounding extrafollicular B-immunoblasts (B-IBs) and Reed-Sternberg-like (RS-like) cells, near epithelioid granulomas, and between the processes of IDCs (Figure 2, Figure 3 and Figure 4).

**Figure 6:**
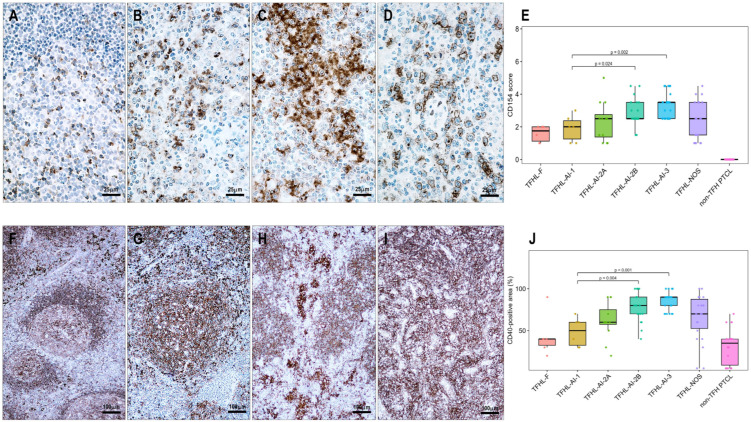
CD154 expression levels in reactive and neoplastic T cells (**A**–**D**). (**A**) Normal CD154 expression in a hyperplastic B-cell follicle. (**B**) TFHL-AI-1, CD154 positivity score = 3 (strong positivity: 3; small cell morphology: 0). (**C**) TFHL-AI-2B, CD154 positivity score = 4.5 (strong staining: 3; medium-sized cells: 0.5; tumor cell-rich pattern: 1). (**D**) TFHL-AI-3, CD154 positivity score = 4 (strong staining: 3; large cell morphology: 1) ((**A**–**D**) magnifications ×400). (**E**) Box plots show the distribution of CD154 positivity scores across lymphoma types, with pairwise comparisons annotated. CD154 positivity score was significantly higher in TFHL-AI-2A than in TFHL-AI-1 (*p* = 0.024) and in TFHL-AI-3 than in TFHL-AI-1 (*p* = 0.002). (**F**–**I**) Distribution of CD40 positivity in reactive and T-cell lymphoma tissue. (**F**) Normal distribution of CD40-positive cells: a hyperplastic lymph node shows CD40 positivity in the mantle zone, germinal centers, and scattered extrafollicular dendritic cells. (**G**) TFHL-AI-1 showing CD40 positivity mainly in the abnormal follicles. The CD40-positive area is 60%. (**H**) TFHL-AI-2B case exhibits CD40 positivity in an abnormal follicle, epithelioid cell granulomas, and extrafollicular dendritic cells. The CD40-positive area is 70%. (**I**) TFHL-AI-3 case displays diffuse CD40 positivity; the densely arranged CD40-positive cellular network is interrupted only by HEVs. The CD40-positive area is 100% (magnification (**F**–**I**) ×100). (**J**) CD40-positive area differed significantly across the examined lymphoma subtypes (*p* < 0.001). Median CD40-positive area was lowest in non-TFH PTCL and TFHL-F, and highest in TFHL-AI-2B and TFHL-AI-3.

Statistically, the CD154 positivity score differed significantly across subtypes (*p* < 0.001). The median CD154 score was 0 in non-TFH PTCL, 1.75 in TFHL-F, 2.0 in TFHL-AI-1, 2.5 in TFHL-AI-2A, 2.5 in TFHL-AI-2B, 3.5 in TFHL-AI-3, and 2.5 in TFHL-NOS. The CD154 positivity score was significantly higher in TFHL-AI-2B than in TFHL-AI-1 (*p* = 0.024) and in TFHL-AI-3 than in TFHL-AI-1 (*p* = 0.002) (Figure 6). The CD154 positivity score was statistically significantly correlated with both CD4 positivity (Fisher’s exact test, *p* < 0.001, odds ratio 940.7, 95% confidence interval 73.5 to 4.50 × 10^15) and the TFH cell phenotype (Fisher’s exact test, *p* < 0.001, odds ratio 40.9, 95% confidence interval 7.07 to 443.3). Multivariable analysis using Firth’s penalized-likelihood logistic regression confirmed the strong associations (odds ratio = 1002.6, 95% confidence interval 76.5 to 156,385.7, *p* < 0.001). However, after adjustment for CD4 positivity, the TFH phenotype was not independently associated with CD154 positivity (odds ratio = 0.37, 95% confidence interval 0.22 to 8.70, *p* = 0.54). The wide confidence intervals and inflated odds ratio estimates are attributable to near-complete separation in the data, indicating an exceptionally strong association rather than inestimability of the effect.

**Figure 7:**
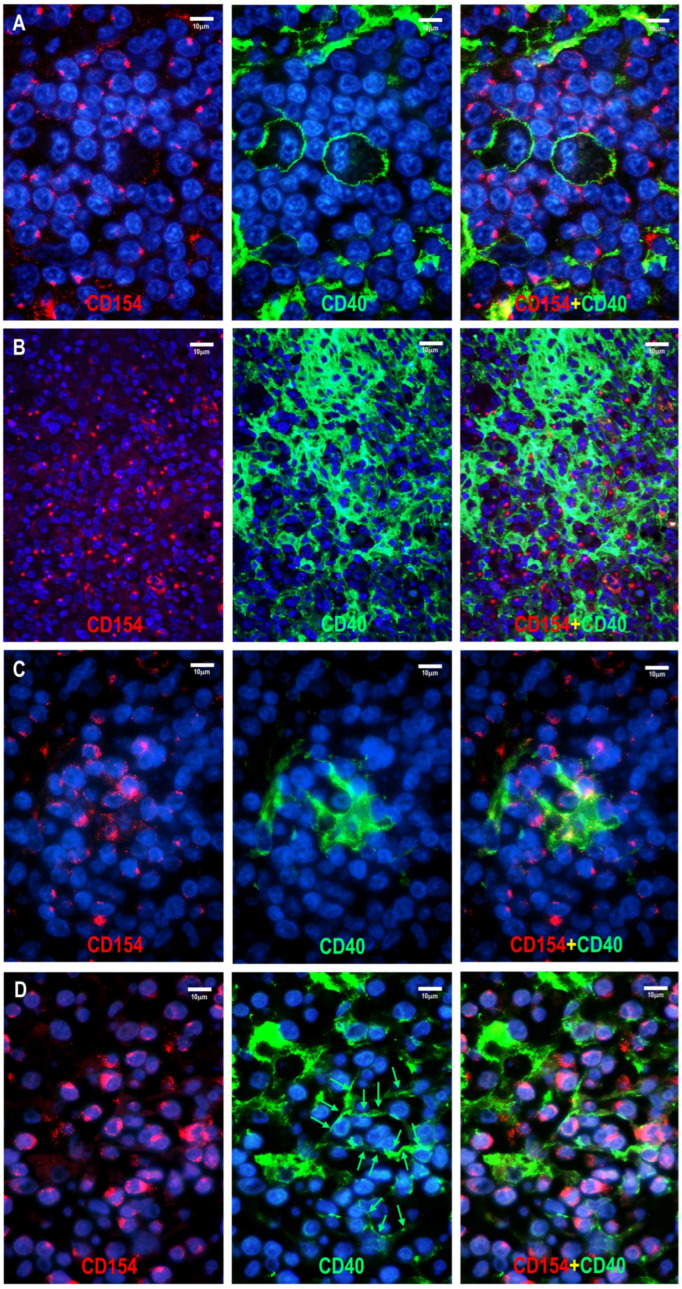
Colocalization of CD154-positive neoplastic T cells with various CD40-positive cells. (**A**) CD154-positive neoplastic T cells surround CD40-positive RS-like cells (multiple immunofluorescence labeling, DAPI nuclear staining, magnification ×1000). (**B**) CD154-positive neoplastic cells in direct contact with the CD40-positive FDC network (multiple immunofluorescence labeling, DAPI nuclear staining, magnification ×400). (**C**) CD154-positive neoplastic T cells in intimate contact with IDC processes (multiple immunofluorescence labeling, DAPI nuclear staining, magnification ×1000). (**D**) CD154-positive neoplastic T cells in close contact with CD40-positive IDCs and FRCs (green arrows) (multiple immunofluorescence labeling, DAPI nuclear staining, magnification ×1000).

The examined T-cell lymphoma cases showed a wide range of CD40-positive cells. B cells, including mantle zone-type small B cells, residual germinal center B cells, and extrafollicular B cells (B-IBs and RS-like cells), displayed moderate to strong CD40 positivity (Figure 2, Figure 3, Figure 4, Figure 5, Figure 6 and Figure 7), whereas plasma cells were negative. Macrophages, epithelioid cells (EpCs), and IDCs consistently exhibited strong CD40 expression. FDCs showed moderate to strong CD40 positivity, whereas FRCs demonstrated weak staining. In contrast, neoplastic TFH cells were consistently CD40-negative. In the TFHL-AI-1 and TFHL-AI-2A cases, CD40 positivity was observed in germinal center B cells, mantle zone B cells, and follicular/perifollicular areas.

Strongly CD40-positive dendritic cells, identical to IDCs, were widely distributed in both follicular and paracortical compartments in nearly all TFHL cases (Figure 4). In TFHL-AI-2B and TFHL-AI-3 subtype cases, the distribution of CD40-positive cells was more extensive, often diffuse (Figure 2, Figure 3, Figure 4, Figure 5 and Figure 6). In TFHL-F and TFHL-NOS cases, a heterogeneous pattern of CD40 expression was observed. In addition to the expansion of the FDC network and increased CD40 positivity of FRCs, accumulation of strongly positive epithelioid cells and IDRCs also contributed to the extended CD40 positivity, whereas the CD40-positive B-cell population was relatively depleted. In cases with diffuse CD40 positivity, CD40-negative neoplastic T cells with abundant cytoplasm were distinctly visible (Figure 3 and Figure 4).

Statistically, the distribution of CD40-positive cells and the CD40-positive area, reflecting CD40-positive cell mass, differed significantly across the examined lymphoma subtypes (*p* < 0.001). The median CD40-positive area was lowest in non-TFH PTCL (35%) and TFHL-F (40%), intermediate in TFHL-AI-1 (50%) and TFHL-AI-2A (60%), and highest in TFHL-AI-2B (80%) and TFHL-AI-3 (90%) (Figure 2, Figure 3, Figure 4 and Figure 6). TFHL-NOS also showed relatively high CD40 expression (median 70%), although with broader dispersion; few cells in monomorphic cases (median 17%), and more widespread positivity in other cases (median 74%). In pairwise comparisons, the CD40-positive area was significantly higher in TFHL-AI-2B than in TFHL-AI-1 (*p* = 0.004) and in TFHL-AI-3 than in TFHL-AI-1 (*p* < 0.001) (Figure 6). CD40 varied in parallel with CD154 across subtypes; higher CD40 expression tended to be associated with higher CD154 expression. In pairwise comparisons, the CD154 positivity score was significantly higher in TFHL-AI-2B than in TFHL-AI-1 (*p* = 0.024) and in TFHL-AI-3 than in TFHL-AI-1 (*p* = 0.003). Using Spearman’s rank correlation to compare median CD40 and CD154 values across subtypes, a very strong positive association between the two markers (rho = 0.964, *p* < 0.001) was found, indicating that subtypes with higher median CD154 expression also showed higher median CD40-positive area (Figure 6). These findings indicate that the more advanced TFHL-AI subgroups exhibit higher CD40 and CD154 expression.

Histiocytes, especially EpCs, ranging from scattered cells to small granulomas, were also highlighted by strong CD40 positivity (Figure 4) in the majority of TFHL cases (73/93, 78%). In TFHL-AI-1 and TFHL-F cases, EpCs were mainly located within and near pathological follicles. In TFHL-AI-2B and TFHL-AI-3 cases, EpCs were primarily found within the abnormal FDC network, whereas in TFHL-AI-2A and TFHL-NOS cases, most EpCs were extrafollicular.

### 3.4. EBV Status and CD40/CD154 Expression

EBV-positive cells were identified in 80% (72/90) of the TFHL cases. In one monomorphic TFHL-NOS case, the neoplastic T cells showed homogeneous EBV positivity, whereas in the others, B cells were EBV-positive, with frequencies ranging from 1% to 30%. The cytomorphological features of EBV-positive B cells ranged from small lymphocytes to RS-like cells. Of the cases tested, 13 had 20% or more EBV-positive cells, many of which showed polymorphic B-cell proliferation. Additionally, EBV-positive diffuse large B-cell lymphoma (DLBCL) was identified in one TFHL case.

**Figure 8:**
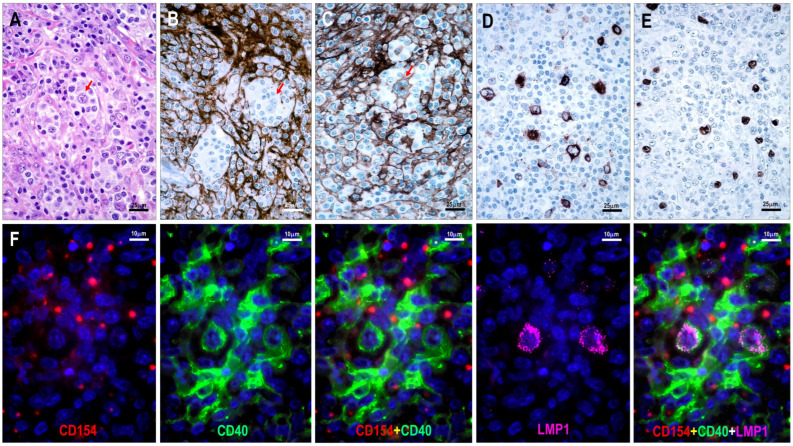
Relationship between CD154, CD40, and EBV-LMP1 expression. (**A**) TFHL-AI-3 case showing large B cells with RS-like morphology (red arrow) surrounded by atypical cells with pale cytoplasm (Hematoxylin and eosin staining). (**B**) CD21-positive dense abnormal FDC network disrupted by a cell cluster containing RS-like cells (red arrow) surrounded by neoplastic T cells. (**C**) CD40-positive diffuse dendritic cell network interrupted by CD40-positive RS-like cell (red arrow) surrounded by neoplastic T cells. Distribution of (**D**) LMP1-positive and (**E**) EBER-positive cells ((**A**–**E**) magnifications ×400). (**F**) TFHL-AI-2B displaying CD154-positive neoplastic cells in close contact with CD40-positive FDCs and transformed large B cells co-expressing CD40 and LMP1 (multiple immunofluorescence labeling, DAPI nuclear staining, ×1000).

**Figure 9:**
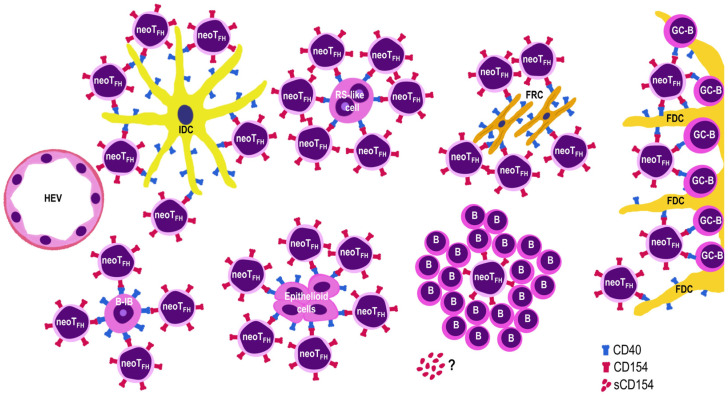
Schematic overview of CD40–CD154 interactions in T follicular helper cell lymphomas. CD154-positive neoplastic T cells (neoTFH), recapitulating interactions of normal TFH cells, bind to each CD40-positive cell in the affected lymph node. These cells include germinal center B cells (GC-B), small B lymphocytes (B), extrafollicular large B cells (B immunoblasts (B-IBs) and Reed-Sternberg (RS)-like cells), interdigitating dendritic cells (IDCs), follicular dendritic cells (FDCs), and fibroblastic reticular cells (FRCs). The CD40–CD154 interaction between these cells mutually promotes their survival and leads to severe immune dysregulation. The question mark indicates that soluble CD154 (sCD154) may also play a role, but this could not be assessed by immunohistochemistry in our study.

Five TFHL cases with high EBV-positive cell counts (1 TFHL-F, 2 TFHL-AI-2B, and 2 TFHL-AI-3) were selected for multiplex immunofluorescence labeling to simultaneously analyze EBV LMP1 and CD40 expression. LMP1 positivity was observed only in a subset of CD40-positive B cells and was primarily found in transformed large B cells with B-IB and RS-like morphology (Figure 8). These cells exhibited reduced CD20 staining intensity and were often surrounded by CD154-positive neoplastic T cells (Figure 9).

Figure 9 provides a schematic overview of findings in TFHLs, illustrating the interaction between CD154-positive neoplastic T cells and CD40-positive cells.

## 4. Discussion

The costimulatory protein CD40 and its ligand, CD154, are essential to the immune system. Their interaction mediates contact-dependent, bidirectional signaling between T and B cells, contributing to germinal center formation, physiological B-cell proliferation and clonal expansion, immunoglobulin production, isotype switching, affinity maturation, and memory B cell development [13,14]. CD40–CD154 crosstalk is crucial for protection against infections and for organ-specific autoimmunity [13,14,24,37]. CD154 is primarily expressed on activated T cells, especially TFH cells, while CD40 is expressed on various cells, including B cells, dendritic cells, macrophages, and fibroblasts [13].

CD40–CD154 interactions have been studied far more extensively in B-cell lymphomas and classical Hodgkin lymphoma [27,38,39] than in T-cell lymphomas [28,35]. Most B-cell lymphomas are CD40-positive, and CD154 plays a multifaceted role in these lymphomas, including aberrant signaling that may contribute to disease progression and resistance to conventional therapies [38,40,41]. In Burkitt lymphoma, the CD40–CD154 interaction influences the survival and differentiation of lymphoma cells. At low concentrations, CD154 can promote cell survival by inhibiting apoptosis, whereas at higher concentrations, it can also induce differentiation [42,43]. In B-CLL and DLBCL, the CD40–CD154 interaction may promote the proliferation and survival of malignant B cells [44]. Although the CD40–CD154 crosstalk is a key costimulatory pathway in T-cell function, in vivo data in human TFHLs are very limited [28,33,34]

TFHLs are characterized by complications arising from disease-related immune dysregulation [1,8,9]. The lymph nodes affected by TFHL exhibit a broad spectrum of morphological alterations across all cellular components, affecting both T- and B-cell compartments. In this study, we found that all (100%) TFHL cases exhibited strong CD154 expression in neoplastic T cells, and observed consistent positivity for CD40 in B cells, follicular dendritic cells, antigen-presenting dendritic cells, fibroblastic reticular cells, and epithelioid histiocytes. Moreover, CD40 expression increased in histologically advanced cases. The high levels of CD154 in tumor cells and increased CD40 expression are consistent with gene-expression profiling data from AITL. Most recently, spatial transcriptome sequencing revealed the role of CD154 in the microenvironmental heterogeneity of AITL [45].

The importance of CD154 in humoral immunity is evident in its deficiency, known as hyper-IgM syndrome [46,47]. These patients fail to form germinal centers and undergo immunoglobulin isotype switching, resulting in recurrent infections [46,47]. Our findings indicate that neoplastic T cells behave like TFH cells, recapitulating their interactions and remodeling the affected lymphoid tissue, thereby causing severe immune dysregulation. According to our interpretative model, in the early phase of the neoplastic process, TFH cell function may be partially preserved, allowing activation of germinal center B cells and leading to hyperplastic follicle formation, as seen in TFHL-AI-1. In certain cases, CD154-positive neoplastic TFH cells accumulate in the follicles but lose their ability to promote B cells to form germinal centers, as demonstrated in TFHL-F. As normal CD40–CD154 function deteriorates, the number of germinal center B cells declines gradually, leading to follicular regression, as seen in TFHL-AI-2. In histologically advanced cases, lymph node tissue undergoes marked transformation, with excessive expansion of CD40-positive FDC networks, leading to severe distortion of the follicular and paracortical compartments, as observed in TFHL-AI-3. Due to the displacement of normal TFH cells, the number of germinal center B cells gradually decreases and inversely correlates with the growth pattern: high in TFHL-AI-1, reduced in TFHL-AI-2, and absent in TFHL-AI-3. Our findings support the conclusion that neoplastic TFH cells disrupt germinal center formation and follicular B-cell development, thereby impairing T-cell-dependent humoral immune responses. Most TFHL cases contained CD40-positive transformed large B cells and RS-like cells, which were in direct contact with or rosetted by CD154-positive neoplastic T cells. These findings suggest that CD40-positive B-IBs and RS-like cells may substitute for depleted germinal center B cells in advanced cases, and that the survival of neoplastic T cells may depend on these cells. This observation is consistent with findings from a mouse model of TFHL, which showed that TET2-mutant B cells and other immune cells promote lymphoma development and the expansion of RHOA G17V-mutant neoplastic TFH cells. The interaction between CD154-positive neoplastic T cells and CD40-positive IDCs, which is consistent with APCs, also contributes to immune dysregulation, leading to immunodeficiency and recurrent infections, common complications in TFHLs. Additionally, CD40 engagement on APCs promotes clonal expansion and survival of effector cells, and this interaction is also implicated in TFHL and may contribute to neoplastic T-cell survival and proliferation. In our TFHL cases, we observed a high number of CD40-positive histiocytes, particularly EpCs, which interacted with CD154-positive neoplastic T cells, suggesting that these cells also play an important role in the disease microenvironment.

CD154 exists in two functional forms: a transmembrane form and a soluble extracellular form [16,20,21]. The latter is derived from the membrane-bound protein after cleavage by ADAM10 and ADAM17 upon engagement with CD40 [48,49]. Similarly, a soluble form of CD40 (sCD40) is shed after proteolytic cleavage of the membrane-anchored CD40 by ADAM17 [50]. Although our evaluation showed that the polyclonal goat anti-CD154 antibody used in this study is specific for the extracellular domain, consistent with sCD154, immunohistochemistry is not reliable for detecting soluble proteins. However, we can assume that sCD40 and sCD154 may be involved in TFHL, further complicating the neoplastic microenvironment.

In addition to the canonical receptor CD40, CD154, especially in its soluble form, can interact with adhesion molecules, including α5β1 [51,52]. T and B cells can express α5β1 on their surfaces [53], and the CD154-α5β1 interaction can prevent Fas-induced T-cell death [54,55] and may also be active in TFHL.

EBV-positive B cells are typically increased in TFHLs, and it has been proposed that EBV infection results from disease-related immunodeficiency [56,57]. EBV load has prognostic significance in TFHL [58]. Polyclonal expansion of B cells or plasma cells, or secondary, often EBV-positive, aggressive B-cell lymphoma may also be part of the disease evolution [1,2,8,57,58,59]. In our cohort, 80% of cases contained EBV-positive cells. The proportion of these cells varied widely, even within the same lymph node and neighboring nodes. In cases studied with multiple labeling, EBV was exclusively present in B cells. Cases with higher EBV levels often showed polymorphic B-cell proliferation, although EBV-negative plasma cell or polymorphic B-cell proliferations also occurred. Only one TFHL case with associated EBV-positive DLBCL was identified, likely due to limited follow-up data. It has been shown that EBV LMP1 acts as a molecular mimic of CD40, substituting for CD40 signaling in B cells and supporting their development, activation, germinal center formation, and, uniquely, isotype switching independently of T-cell help [60,61,62,63]. In our cases, LMP1-positive B cells co-expressed CD40, supporting the idea that both LMP1 and the CD40–CD154 interaction may actively promote B cell survival and transformation, and consequently, the survival of neoplastic T cells. However, the number of LMP1-positive cells was often lower than that of EBER-positive cells, suggesting that CD154 can substitute for LMP1 in B-cell transformation, as described with the LMP1-deficient EBV mutant [64]. The cooperation of the CD40–CD154 axis and EBV may serve as an additional driver of disease evolution and the development of secondary B-cell lymphomas.

TFHL represents an aggressive, frequently relapsing mature T-cell lymphoma entity with a poor prognosis and a 5-year survival of approximately 30%. Conventional first-line treatment typically involves anthracycline-based chemotherapy, CHOP (cyclophosphamide, doxorubicin, vincristine, prednisone) or CHOEP (with etoposide), followed by consolidation autologous stem cell transplantation [8,9]. Contemporary therapeutic strategies have not yet led to significant improvements over chemotherapy; therefore, novel treatment approaches are needed [9]. Our findings suggest that neoplastic T-cell proliferation in TFHL correlates with higher levels of CD154 and CD40 expression, potentially influencing the disease evolution. Consequently, targeting the CD40–CD154 interactions may offer a valuable approach for TFHL treatment. 

In many autoimmune diseases, including systemic lupus erythematosus, autoimmune arthritis, and myasthenia gravis, blocking the CD40–CD154 pathway has been shown to prevent or suppress the disease [37,65,66,67,68]. Inhibiting the CD40–CD154 interaction appears promising for preventing organ transplant rejection [69,70,71]. In B-cell lymphomas, CD40 antagonists have shown promise [52,72]. In mouse models, administration of anti-CD154 antibodies has been shown to extend survival [31]. In humans, early attempts to utilize humanized anti-CD154 antibodies in autoimmune diseases have led to serious thrombotic complications because CD154 is expressed on platelets [19]. Nevertheless, newer CD154 agents are on the horizon that may help reduce side effects [37,73,74].

## 5. Conclusions

To the best of our knowledge, this is the first comprehensive immunomorphological study conducted in humans to reveal the significance of CD40–CD154 interaction in TFHL. Here, we demonstrate that neoplastic T cells in TFHLs consistently express CD154, while nearly all other cells in the affected lymph nodes express CD40. The CD154-positive neoplastic T cells recapitulate the intranodal migration of normal TFH cells, remodeling each functional compartment. This explains the complex microenvironmental alterations and immune dysregulation related to the disease. The colocalization between CD154-positive neoplastic T cells and CD40-positive immune cells effectively demonstrates that the CD40–CD154 interaction also occurs in TFHL. This interaction may contribute to disease development and possibly progression, and may offer a promising target for future therapies. Additional research is needed to examine the roles of soluble CD40 and CD154 in the disease process and to identify biomarkers linked to CD40–CD154 signaling.

## Figures and Tables

**Table 1:** Summary of CD154 positivity in the studied PTCL cases.

Diagnosis	No of Cases	CD154 Positivity
TFHL-AI-1	6	100% (6/6)
TFHL-AI-2	32	100% (32/32)
TFHL-AI-3	26	100% (26/26)
TFHL-F	6	100% (6/6)
TFHL-NOS	23	100% (23/23)
TFHL (overall)	93	100% (93/93)
non-TFH PTCL	18	6% (1/18)

## Data Availability

The data presented in this study are available on request from the corresponding author. The data are not publicly available due to ethical restrictions.

## Supplementary Materials

The following supporting information can be downloaded at: https://www.mdpi.com/article/10.3390/cells15090785/s1, Table S1: Summary of demographic and pathological data for the analyzed cases; Table S2: List of antibodies used in the study.

## Abbreviations

AITL	Angioimmunoblastic T-cell lymphoma
APC	Antigen-presenting cell
BCL6	B-cell lymphoma 6
B-IB	B immunoblast
CXCL13	CXC chemokine ligand 13
DLBCL	Diffuse large B-cell lymphoma
EBER	EBV-encoded small RNA
EBV	Epstein–Barr virus
EpC	Epithelioid cell
FDC	Follicular dendritic cell
FRC	Fibroblastic dendritic cell
HEV	High endothelial venule
ICOS	Inducible T-cell costimulatory
IDC	Interdigitating dendritic cell
LMP1	EBV-encoded latent membrane protein 1
PD1	Programmed cell death protein 1
PTCL	Peripheral T-cell lymphoma
RS-like	Reed-Sternberg-like
T_FH_	T follicular T helper
TFHL	T follicular T helper cell lymphoma
TFHL-AI	T follicular T helper cell lymphoma angioimmunoblastic-type
TFHL-AI-1	T follicular T helper cell lymphoma angioimmunoblastic-type, pattern 1
TFHL-AI-2	T follicular T helper cell lymphoma angioimmunoblastic-type, pattern 2
TFHL-AI	T follicular T helper cell lymphoma angioimmunoblastic-type, pattern 3
TFHL-F	T follicular T helper cell lymphoma follicular-type
TFHL-NOS	T follicular T helper cell lymphoma not otherwise specified-type
