# Supplementary material for: Immunomorphological Analysis of the CD40–CD154 Interaction in T Follicular Helper Cell Lymphoma Emphasizes the Significance of the CD40–CD154 Axis in the Disease
Source: Cells. 2026 Apr 26;15(9):785. doi: 10.3390/cells15090785 (PMC13162781; doi:10.3390/cells15090785)
Supplement: Supplementary file 1 [file cells-15-00785-s001.zip › Supplementary Table S2.pdf]

Supplementary Table S2. *List of antibodies used in the study*

**Antibody/Clone**	**Origin**	**Source**
BCL6/LN22	Mouse	Leica/Novocastra
CD3/H-12	Mouse	Santa Cruz Biotechnology
CD4/N1GU0	Mouse	ThermoFisher/eBioscience
CD5/polyclonal	Rabbit	ThermoFisher/Invitrogen
CD8/C8/144B	Mouse	Santa Cruz Biotechnology
CD10/56C6	Mouse	Leica/Novocastra
CD20/L26	Mouse	Leica/Novocastra
CD21/ER3093	Rabbit	Histopathology*
CD40/CL1673	Mouse	Bio-techne/NovusBio
CD40/Polyclonal	Rabbit	ThermoFisher/Invitrogen
CD134/H10	Mouse	Santa Cruz Biotechnology
CD154/1E6D10	Mouse	Proteintech
CD154/polyclonal	Goat	Bio-techne/R&D Systems
CXCL13/polyclonal	Goat	Bio-Techne/R&DSystems
CXCR5/polyclonal	Rabbit	Atlas Antibodies
CXCR5/51505	Mouse	Bio-Techne/R&DSystems
EBV-LMP1/CS1-4	Mouse	Agilent/DAKO
ICOS/D1K2T	Rabbit	Cell Signaling Technology
ICOS/polyclonal	Goat	Bio-Techne/R&DSystems
PD1/polyclonal	Goat	Bio-Techne/R&DSystems
TIA-1/ 2G9A10F5	Mouse	Beckam-Coulter Life Sciences

*Histopathology Ltd., Hungary
